# Author Correction: Effect of sub-dermal exposure of silver nanoparticles on hepatic, renal and cardiac functions accompanying oxidative damage in male Wistar rats

**DOI:** 10.1038/s41598-023-38492-0

**Published:** 2023-07-12

**Authors:** Janet Olayemi Olugbodi, Bashir Lawal, Godiya Bako, Amos Sunday Onikanni, Sulama M. Abolenin, Soliman S. Mohammud, Farid S. Ataya, Gaber El-Saber Batiha

**Affiliations:** 1 Department of Biochemistry, Bingham University, Abuja-Keffi Expressway Road, P.M.B 005, Karu, Nigeria; 2 Department of Pathology, University of Pittsburgh, Pittsburgh, United States; 3 UPMC Hillman Cancer Center, University of Pittsburgh, Pittsburgh, United States; 4 Biochemistry Unit, Department of Chemical Sciences, Afe Babalola University, Ado-Ekiti, Ekiti State Nigeria; 5 College of Medicine, Graduate Institute of Biomedical Science, China Medical University, Taichung, Taiwan; 6 Biology Department, Thurobah University College, Thurobah, Republic of Congo; 7 Department of Biochemistry, College of Science, King Saud University, P. O. Box 2455, 11451 Riyadh, Saudi Arabia; 8 Department of Pharmacology and Therapeutics, Faculty of Veterinary Medicine, Damanhour University, Damanhour, 22511 AlBeheira Egypt

Correction to: *Scientific Reports* 10.1038/s41598-023-37178-x, published online 29 June 2023

The original version of this Article contained errors in Figure 8, where panel (**E**) was a duplication of panel (**F**) for ‘heart’. The original Figure 8 and accompanying legend appear below.

**Figure 8:**
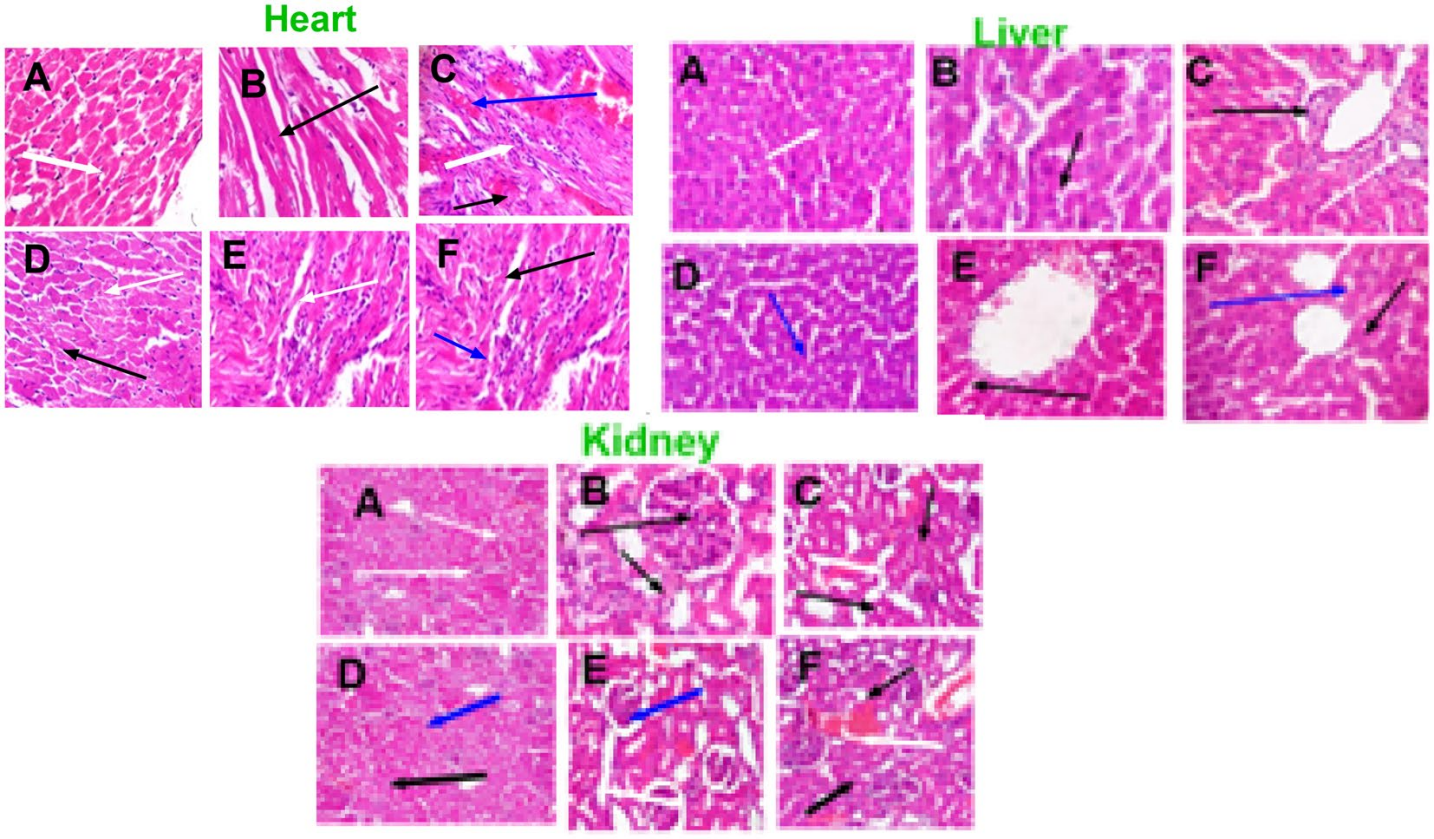
Histopathological analysis of the liver, heart and kidney of rats administered with AgNPs after 14 and 28 days. (**A**) Control (14 days exposure), (**D**) Control (28 days exposure) (**B**) (10 mg/kg AgNPs; 14 days exposure), (**E**) (10 mg/kg AgNPs; 28 days exposure), (**C**) (50 mg/kg AgNPs; 14 days), (**F**) (50 mg/kg AgNPs; 28 days exposure) H&E Staining, Magnification × 400.

The original Article has been corrected.

